# Acute Effects of Immersive Virtual Reality Exercise on Young Adults’ Situational Motivation

**DOI:** 10.3390/jcm8111947

**Published:** 2019-11-12

**Authors:** Wenxi Liu, Nan Zeng, Zachary C. Pope, Daniel J. McDonough, Zan Gao

**Affiliations:** 1 School of Kinesiology, University of Minnesota-Twin Cities, Minneapolis, MN 55455, USA; liux4443@umn.edu (W.L.); mcdo0785@umn.edu (D.J.M.); 2 Department of Food Science and Human Nutrition, Colorado State University, Fort Collins, CO 80523, USA; nanzeng@colostate.edu; 3 School of Public Health, University of Minnesota-Twin Cities, Minneapolis, MN 55455, USA; popex157@umn.edu

**Keywords:** virtual reality, physical activity, motivation, college students

## Abstract

The development of innovative technology, such as virtual reality (VR), has provided opportunities for promoting physical activity (PA) in a fun and engaging manner. The purpose of this study was to examine differences in young adults’ situational motivation (SM) among immersive VR, non-immersive VR, and traditional stationary cycling sessions. In all, 49 healthy college students (35 females; M*_age_* = 23.6 years, SD = 3.4; M_%BF_ = 24.0%, SD = 7.5) completed three separate 20 min cycling sessions: (1) immersive VR cycling; (2) non-immersive VR cycling; and (3) traditional cycling. Participants’ SM was assessed via the situational motivation scale, which included four subconstructs: intrinsic motivation, identified regulation, external regulation, and amotivation. Repeated measures ANOVAs indicated significant differences for situational motivation between cycling sessions (*F* (2, 96) = 4.74–53.04, *p* < 0.01, *η_p_*^2^ = 0.090–0.525). Specifically, participants elicited the highest level of intrinsic motivation in immersive VR cycling compared to the other two sessions. Moreover, participants in both immersive VR and traditional cycling showed greater identified regulation than the non-immersive VR session. Furthermore, participants showed greater external regulation compared to the immersive VR session. In addition, greater amotivation was observed in non-immersive VR compared to the immersive VR session. Findings suggested that immersive VR exercise has the potential to be an attractive exercise alternative, possibly promoting greater PA participation and adherence among young adults.

## 1. Introduction

Despite the known health benefits of regular physical activity (PA) participation, physical inactivity among U.S. adults remains a major public health concern [1]. Young adults, such as college students, are a population at risk for decreased PA participation due to the newfound responsibility of balancing school, work, and personal responsibilities. According to the National College Health Assessment (NCHA), only 22.7% and 18.6% of male and female college students, respectively, meet recommended guidelines for moderate-intensity PA of ≥30 min 5–7 days each week in 2018 [2]. Generally, college students have most often reported campus recreation center quality, lack of time or motivation, and the need for social support to engage in PA as impeding their regular PA participation [3,4]. The NCHA also reported that >40% college students were overweight or obese in 2018 [2]. This observation was notable as overweight and obesity in young adulthood has been found to track into later life [5,6]. Previous research has indicated that many adult health behaviors are developed and established during late adolescence and early adulthood. It is, therefore, imperative to examine innovative ways to motivate college students to participate in regular PA to improve physical and psychological well-being in this population [7,8,9].

The young adult population is technology-savvy and interested in videogame play. According to the Pew Research Center, among college students, 70% reported playing videogames, computer games, or online games at least once a week. Moreover, approximately 65% of college students reported being regular or occasional game players—possibly contributing to increased sedentary time [10]. Virtual reality (VR)-based exercise is an active form of videogame-based technology potentially attractive to the college population as a means of promoting regular PA participation. VR is an interactive computer-generated experience taking place within a simulated environment, with this technology primarily incorporating auditory and visual feedback [11]. There are two types of VR technology—immersive VR and non-immersive VR. In detail, immersive VR utilizes a head-mounted apparatus, body motion sensor, real-time graphics, and an advanced interface device to simulate the complete virtual environment, which envelopes players in a virtual world. Non-immersive VR uses a flat computer/television screen linked to a keyboard, gamepad, and joystick to interact with the gaming system [12], and is sometimes referred to as exergaming or active video games [13,14,15]. Immersive VR makes players feel like they are “actually in the virtual world”, whereas non-immersive VR does not simulate a virtual environment to a deeper degree [16,17]. 

Currently, VR technology has been primarily used as a rehabilitation tool among clinically based applications, such as patients with phobias and Parkinson’s disease [18,19,20,21]. For example, phobia patients in VR exposure therapy showed greater efficacy when compared to an imaginary exposure [22]. The underlying mechanism may be due to exposure to emotional situations and prolonged rehearsal leading to the regular activation of cerebral metabolism in brain regions, which was associated with inhibition of maladaptive associative processes [23]. However, there is a paucity of literature regarding the implementation of VR technology on PA promotion in healthy populations. Recently, researchers and health professionals have begun to explore the feasibility and effectiveness of employing VR-based exercise as an innovative health promotion approach. Indeed, Blascovich et al. promoted the use of immersive virtual environment technology as a methodological tool in psychology research [24]. In addition, Zeng et al. recently conducted a pilot study that compared an immersive VR-based exercise bike and a traditional exercise bike on physiological and psychological responses [11]. The study found participants during VR-based exercise had significantly higher enjoyment and self-efficacy compared to traditional stationary bike exercise, suggesting VR-based exercise to be an effective, enjoyable, and motivating tool for promoting PA among adult populations. Although Zeng’s study demonstrated the enjoyable nature of VR-based exercise, it is unclear what motivation participants experienced when they were engaging in VR-based exercise. Therefore, based on this preliminary evidence, the present study aimed to investigate the effects of VR-based exercise bike on college students’ situational motivation (SM).

The use of VR technology as a health promotion tool may serve to increase college students’ motivation to engage in PA. Research on college students’ PA motivation has revealed the degrees of self-determined motivation when participating in PA [25,26,27,28]. According to the self-determination theory, an individual’s motivation is categorized into levels of higher to lower self-determination, namely, intrinsic motivation, identified regulation, external regulation, and amotivation [29,30,31]. Intrinsic motivation is present when behavior is intrinsically motivated by experiencing satisfaction and pleasure. Identified regulation occurs when a behavior is valued and perceived as being chosen by oneself. In contrast, external regulation is present when an individual engages in a behavior to obtain rewards or avoid punishment. Finally, when amotivation occurs, individuals experience a lack of contingency between their behaviors and outcomes. College students are more likely to participate and adhere to a certain activity if they want to do it for their own sake, which refers to intrinsic motivation [32]. Therefore, examining and promoting motivation is crucial when implementing a new PA intervention. Recently, Kim et al. conducted an experimental study evaluating the effectivenss of immersive VR cycling exercise (VirZoom VR bike) on health-related PA in comparison to traditional stationary cycling [33]. In their study, they found participants on immersive VR cycling experienced more arousal and motivation compared to stationary cycling. As such, it was suggested that VR may directly influence players’ psychological or physiological states in addition to the extent to which they feel present in the virtual environment [30,31]. However, the previous study did not specify which type of self-determined motivation resulted in those positive outcomes. In order to develop an effective VR-based exercise program, it is important to fully understand different types of motivation influencing individuals’ psychological and physiological states. While intrinsic motivation has been well documented as a determinant of increased regular PA participation, it is unknown whether other types of motivation, such as identified regulation, external regulation, and amotivation, play a role in improving motivation for PA during participation in technology-based exercise modalities, such as VR. Thus, we conducted the current study to investigate the effects of immersive VR cycling exercise on college students’ SM, compared to non-immersive VR cycling exercise and traditional cycling exercise. We hypothesized that participants would have higher levels of intrinsic motivation during immersive VR cycling compared to non-immersive VR cycling and traditional cycling sessions. Moreover, we hypothesized that immersive VR cycling would have higher identified regulation than non-immersive VR cycling and traditional cycling sessions. Lastly, we hypthesized that immersive VR cycling would have the least external regulation and amotivation compared to the other two cycling sessions. Study observations may provide preliminary evidence to researchers and health professionals regarding the feasibility and effectiveness of VR-based exercise for PA and health promotion.

## 2. Materials and Methods

### 2.1. Participants

In all, 49 healthy college students were recruited from an urban public university in the Midwest region of the U.S. The inclusion criteria were as follows: (1) 18–35 years old; (2) no self-reported diagnosed physical or mental disability; (3) successful completion of Physical Activity Readiness Questionnaire [34]; and (4) provision of informed consent for participation. Participants successfully completed the three cycling sessions and measurements with no reported motion sickness or visual fatigue. This study was approved by the University Institutional Review Board (number: 1703M10342, 04/27/2017) prior to any data collection. All procedures were performed in accordance with the ethical standards of the Institution and/or national research committee and with the 1964 Helsinki Declaration and its later amendments or comparable ethical standards [35]. 

### 2.2. Research Design and Settings

The present study was a cross-sectional design, with data collected in a well-established and highly controlled laboratory. Participants completed three separate 20 min exercise sessions in a counterbalanced order: (1) immersive VR cycling (VirZoom Bike connected to a PlayStation 4; Cambridge, MA, USA; Sony; Tokyo, Japan); (2) non-immersive VR cycling (Gamercize Bike connected to an Xbox 360; Southampton, UK; Microsoft; Redmond, WA, USA); and (3) traditional cycling (Spirit Fitness 156 XBU55 Upright Bike; Spirit Fitness; Jonesboro, AR, USA). 

### 2.3. Immersive VR Cycling Session

The commercially available VirZoom VR exercise bike offers players a fully immersive virtual gaming environment via connection of the VirZoom bike to a PlayStation VR system and an accompanying VR headset. Participants played two games during the 20 min exercise session. The first game was entitled “Le Tour” which required players to race other virtual cyclists through a series of timing gates located on a scenic mountain road. The second game, “Race Car”, required players to race other virtual race cars around a track at high speeds [11]. Both games required participants to pedal faster or slower for speeding up or slowing down, with the leaning of the torso side-to-side allowing participants to turn left or right during gameplay. 

### 2.4. Non-Immersive VR Cycling Session

The Gamercize exercise bike was used during the non-immersive VR condition and was connected to the Xbox 360. Participants played “Motocross”, where players maneuver through a racecourse against virtual competitors in the game. A standard Xbox 360 controller allowed participants to steer the motocross bike during the game. Importantly, participants had to keep their pedaling cadence above 60 rotations per minute, otherwise the Xbox 360 controller would shut off and stop gameplay.

### 2.5. Traditional Cycling Session

The Spirit Fitness XBU55 upright stationary bike was used for the traditional cycling session. The immersive VR and non-immersive VR cycling sessions were observed to be equal to 65% to 85% of age-predicted maximum heart rate. To ensure exercise intensities were comparable among three cycling sessions, participants were also required to maintain their heart rate between 65% and 85% of age-predicted maximum heart rate, which was assessed by using the bike’s built-in heart rate monitor during the traditional cycling session. 

### 2.6. Procedure

Prior to participating in the three cycling sessions, participants’ height, weight, and body fat percentage were assessed, and demographic information was also gathered. Participants then completed each session in a counterbalanced order. Breaks between each cycling session were approximately 10 min in duration and allowed for participants’ blood pressure and heart rate to return to baseline levels—as suggested in other exercise literature [36]. During these between-session breaks, participants were asked to complete the SM survey. Participants received a $20 incentive after successfully completing all the sessions. 

### 2.7. Demographic and Anthropometric Information

Participants’ age, sex, and race/ethnicity were obtained from a self-reported demographic questionnaire. Height was measured to the nearest 0.5 cm using a Seca stadiometer (Hamburg, Germany). Weight and body fat percentage were assessed via the Tanita BC-558 IRONMAN^®^ Segmental Body Composition 164 Monitor (Tokyo, Japan). The body mass index (BMI) was calculated by the weight divided by the square of height (kg/m^2^) [37]. 

### 2.8. SM

A validated 16-item SM survey with a 7-point Likert-type scale (1 = strongly disagree; 7 = strongly agree) was used to assess participants’ SM [32]. The scale was constructed based upon self-determination theory (SDT), and included four subscales corresponding to intrinsic motivation, identified regulation, external regulation, and amotivation [38]. Each subscale included a 4-item survey. The sample item for intrinsic motivation included “Because I think this activity is interesting”; the identified regulation sample item included “Because I think that this activity is good for me”; the external regulation sample item included “Because I am supposed to do it”; and the amotivation sample item included “I do this activity but I am not sure if it is worth it”. Participants were required to complete this survey immediately following each cycling session. Each subscales’ mean scores were calculated and used as the primary outcomes. Specifically, higher scores on intrinsic motivation and identified regulation indicated that participants had higher levels of self-determined motivation. Inversely, higher scores on external regulation and amotivation indicated that participants had lower self-determined motivation for engaging in such types of activity. 

### 2.9. Statistical Analyses 

First, data were screened for the outliers using a boxplot and normality of distributions using the Shapiro–Wilks tests of normality. Second, descriptive statistics were used to describe participants’ characteristics and study outcomes. Third, repeated measures ANOVAs were used to examine the differences in SM (intrinsic motivation, identified regulation, external regulation, and amotivation) among three cycling sessions. In addition, the confidence interval (CI) was reported to indicate the magnitude of difference between sessions. All analyses were performed using IBM SPSS 25.0 (Armonk, NY, USA). The significant level was set to *p* < 0.05. 

## 3. Results

A total of 49 college students (35 females; M*_age_* = 23.6 years, SD = 3.4; M_BMI_ = 23.8, SD = 3.6; M_%BF_ = 24.0%, SD = 7.5) completed three cycling sessions, with no motion sickness or visual fatigue during VR sessions reported. Participant characteristics are presented in Table 1. In addition, Table 2 displays mean scores for each SM subscale. Repeated measures ANOVAs revealed significant differences between the three cycling sessions for intrinsic motivation (*F* (2, 96) = 53.04, *p* < 0.01, *η_p_*^2^ = 0.525), identified regulation (*F* (2, 96) = 11.51, *p* < 0.01, *η_p_*^2^ = 0.193), external regulation (*F* (2, 96) = 4.74, *p* = 0.01, *η_p_*^2^ = 0.090), and amotivation (*F* (2, 96) = 7.65, *p* = 0.01, *η_p_*^2^ = 0.137). Post hoc analysis indicated that participants had higher levels of intrinsic motivation during immersive VR cycling compared to non-immersive VR cycling (95% CI: 0.89, 1.83) and traditional cycling (95% CI: 1.79, 3.05), with participants also having higher levels of intrinsic motivation during non-immersive VR cycling compared to traditional cycling (95% CI: 0.42, 1.70). Moreover, participants had higher levels of identified regulation during immersive VR cycling compared to non-immersive VR cycling (95% CI: 0.34, 0.92), and participants reported lower levels of identified regulation in non-immersive VR cycling compared to traditional cycling (95% CI: −0.70, −0.02). Participants reported lower levels of external motivation during immersive VR cycling compared to traditional cycling (95% CI: −0.85, −0.10). Lastly, participants reported lower levels of amotivation during immersive VR cycling in comparison to non-immersive VR cycling (95% CI: −1.04, −0.21).

## 4. Discussion

The present study examined the differences of SM (intrinsic motivation, identified regulation, external regulation, and amotivation) between immersive VR cycling, non-immersive VR cycling, and traditional cycling sessions. Based on the principles of SDT and previous VR research, we hypothesized that we would observe significant differences between the three cycling sessions, with VR cycling resulting in higher levels of self-determined motivation. Indeed, the present study results indicated significant differences of SM observed between the three cycling sessions. In detail, consistent with our original hypothesis, immersive VR cycling promoted greater intrinsic motivation and identified regulation compared to the other two cycling sessions. In addition, participants reported lower external regulation during immersive VR cycling compared to traditional cycling, but there was no significant difference between immersive VR and non-immersive VR cycling. Lastly, participants had lower amotivation during immersive VR cycling compared to non-immersive VR cycling but there was no significant difference when immersive and non-immersive VR were compared to traditional cycling. Overall, the promising findings of this study provided preliminary evidence regarding the effectiveness of using immersive VR cycling as a motivational tool for promoting PA.

Participants in the immersive VR cycling session elicited the highest level of intrinsic motivation, followed by non-immersive VR cycling and the traditional cycling session. The feelings of pleasure or enjoyment arising from participants in VR-based exercise has been considered a potential manner by which to promote PA participation [9,39,40,41]. Based on SDT, enjoyment is positively correlated with intrinsic motivation. In other words, individuals will be more intrinsically motivated when they feel more enjoyment [42]. In the present study, participants reported greater intrinsic motivation during both immersive and non-immersive VR cycling. When comparing the intrinsic motivation between immersive VR and non-immersive VR, we should consider the degree to which those two types of VR bring participants’ the feeling of “presence” in the virtual environment. For example, immersive VR provides visual, audio, and motion sensory feedback which fully immerses participants in the virtual environment. The non-immersive VR provides less sense of immersion when compared to immersive VR. One recent study adopted VR-based exercise for examining the effects of immersion on players’ motivation and exercise performance and found that increased levels of immersion led to increased levels of motivation for engaging in exercise [43]. This may explain our observations of participants’ greater intrinsic motivation during immersive VR compared to non-immersive VR cycling. Previous studies have suggested intrinsic motivation to lead to positive outcomes, such as improved PA, positive affect, and vitality [27]. For instance, Gao et al. examined college students’ SM and effort/persistence in PA participation and found that both intrinsic motivation and identified regulation were significant positive predictors of PA effort/persistence [44]. Corresponding to the current study’s findings, immersive VR cycling exercise may promote greater levels of intrinsic motivation and may therefore motivate future PA participation. In addition, Mestre et al. compared indoor cycling exercise with VR feedback versus non-VR feedback and found that cycling exercise with VR feedback had a reduced perceived exertion and an increased level of enjoyment of PA [45]. Those intrinsically motivated behaviors were usually characterized with enjoyment and satisfying. In other words, participants who engaged in the VR-based exercise may feel more intrinsically motivated than during traditional exercise because they experienced more enjoyment or fun during this activity, which may potentially increase PA participation and decrease sedentary behaviors. Therefore, the current study’s findings suggested immersive VR cycling exercise could be a motivating tool for promoting greater intrinsic motivation of PA participation among college students. 

In addition, we observed participants in both immersive VR and traditional cycling sessions to have higher levels of identified regulation compared to non-immersive VR exercise. According to SDT, identified regulation occurs when a behavior is valued and perceived as being chosen by oneself [32]. It is a relatively higher level of self-determined extrinsic motivation, compared to external regulation and amotivation. Both intrinsic motivation and identified regulation were considered higher self-determined motivation, which usually leads to positive consequences. A previous study has indicated that intrinsic motivation was positively associated with identified regulation but negatively associated with external regulation and amotivation [46]. As expected, we observed higher identified regulation during the immersive VR cycling session versus the non-immersive VR cycling session. The greater immersion and embodiment in the fully immersive environment may have led to greater self-determined motivation in comparison to non-immersive VR cycling. As mentioned, the more the participants experience the sensation of being “present” in the virtual environment, the greater the level of motivation the participants will experience for engaging in PA. As participants perceived or valued themselves to be more involved in the virtual gaming environment, they might be more self-motivated for performing this immersive VR-based exercise modality. 

Moreover, we observed participants in the immersive VR cycling session to have lower levels of external regulation compared to the traditional cycling session. According to the continuum of SDT, both intrinsic motivation and identified regulation refer to higher levels of self-determined motivation and lead to positive consequences, whereas external regulation and amotivation represent lower levels of self-determined motivation and result in negative consequences [27,29]. Participants in the traditional cycling session might feel the external regulation to do such activity with less self-determined motivation involved. Because external regulation emphasizes external reward or punishment rather than the activity itself, participants might perceive external pressure to engage in a given activity, rather than engaging in an activity for their own sake. Notably, participants in the non-immersive VR cycling session had higher levels of amotivation than in the immersive VR cycling session. Not surprisingly, participants were more motivated in the immersive VR cycling than in the non-immersive VR cycling session. A previous motivation study has indicated that intrinsic motivation is negatively associated with amotivation [46]. Participants in the present study had higher levels of intrinsic motivation in playing immersive VR cycling compared to non-immersive VR cycling, and indeed, we observed lower levels of amotivation in the immersive VR cycling session. It seems that participants were more motivated by immersive VR cycling than non-immersive VR cycling. This is promising since college students are more willing to experience the most advanced technologies than the old ones. As such, it seems encouraging and feasible to incorporate the advanced technology, such as immersive VR technology, for promoting PA participation in this population. 

Taken together, these observations provide preliminary empirical evidence that a commercially available VR system (PlayStation 4 VR) along with a VR-based exercise apparatus (VirZoom VR exercise bike) may be feasible to reach a broader population of individuals to promote PA participation and obtain positive health outcomes. To the best of our knowledge, this is the first study examining acute effects of a commercially available VR cycling exercise on college students’ SM compared to non-immersive VR and traditional cycling sessions. The present study’s findings provided the empirical evidence supporting the effectiveness of a commercially available immersive VR cycling exercise (VirZoom) as a motivational tool for promoting PA participation among college students. Nevertheless, the limitations of the current study need to be noted. First, the mostly female and non-Hispanic White sample hinders the ability to identify sex and/or racial differences in outcomes. Future studies need to consider sex and race/ethnicity differences in motivation when aiming to promote PA among young adults. Second, as the participants were young adults in this study, the results may not be generalized to the other populations. Third, the exercise bike’s built-in heart rate monitor may be less accurate than professional heart rate monitors for measuring PA intensities between sessions. Future studies should precisely measure heart rate to ensure the PA intensities are comparable between sessions. Fourth, we observed higher identified regulation in the traditional cycling compared to the non-immersive cycling session. Although the current study used a counterbalancing strategy (with 10 min breaks between sessions) to avoid potential exercising bias, the randomized session placement may still influence participants’ perception of session activities. For instance, after experiencing immersive VR cycling and traditional cycling sessions, participants in the non-immersive cycling session may perceive less enjoyment compared with more technology advanced immersive VR cycling and less perception of health benefits than traditional cycling. Future studies must consider the carryover effects when it requires participants to go through different sessions in a certain time frame. Notably, although our study provided empirical evidence for using immersive VR exercise as a motivational tool PA participation, it is not clear whether the immersive VR-based exercise can fully serve as a substitute for traditional PA for the total amount of time required by the PA guidelines. Thus, future longitudinal studies are needed for investigating long-term effects on physiological and psychological health-related outcomes.

## 5. Conclusions

The findings suggested immersive VR cycling could be a motivating and enjoyable tool for promoting PA participation and PA adherence among college students. VR-based exercise seems feasible to be implemented for PA and health promotion in college populations. Nevertheless, more studies are warranted to further confirm the effectiveness of VR-based exercise in promoting PA participation over the long term and obtain positive health benefits among various populations. 

## Figures and Tables

**Table 1:** Descriptive statistics for participants’ characteristics.

	Mean	SD
Age (years)	23.61	3.39
Height (m)	1.69	0.80
Weight (kg)	68.25	13.90
BMI	23.81	3.56
%BF (%)	23.97	7.46

Note: SD, standard deviation; BMI, body mass index; %BF, percent of body fat.

**Table 2:** Descriptive statistics for situational motivation (SM) outcomes.

	Intrinsic Motivation		Identified Regulation		External Regulation		Amotivation	
	Mean	SD	Mean	SD	Mean	SD	Mean	SD
Immersive VR	6.31	0.72	6.03	0.79	2.72	1.44	1.60	0.74
Non-immersive VR	4.95	1.54	5.40	1.13	2.93	1.55	2.22	1.19
Traditional Cycling	3.89	1.69	5.76	1.21	3.20	1.42	1.86	0.97

Note: SD, standard deviation; VR, virtual reality.
